# Enhanced Vulnerability of Asylum Seekers in Times of Crisis

**DOI:** 10.1007/s12142-023-00690-8

**Published:** 2023-06-12

**Authors:** Stephen Phillips

**Affiliations:** Institute for Human Rights, Åbo Akademi University, Vänrikinkatu 3 A, 20500 Turku, Finland

**Keywords:** Vulnerability, Asylum seekers, Crisis, Exclusion

## Abstract

This article examines the impact of law and policy changes enacted in times of crisis on asylum seekers, and considers the extent to which considerations of vulnerability have played a part in the various approaches of governments. What emerges is a shift towards further exclusion, and a widening divide between how states approach citizens versus others. The result is enhanced vulnerability, and an environment in which the utility of the vulnerability concept to provide the necessary levels of support and protection is tested. By looking at how vulnerability is used by states, and contrasting this use with how the concept is often used by other community actors, the article asks what role the concept of vulnerability might play in the effects of crises on vulnerable groups and the priorities and actions of states.

## Introduction

People, societies, and governments react to crises in different ways, and with various outcomes. One feature of crisis is a sense of uncertainty, of unpredictability, and that a response must eventuate to restore a sense of order and control. The implications for people following these responses to crisis vary depending on the position of each individual and group, especially where governments and other actors act in haste, or certain agendas receive priority. Asylum seekers are widely recognized as a particularly vulnerable group in need of special protection (Freedman 2019; Freier et al. 2022). In times of crisis, vulnerability can increase and intensify (Suryahadi and Sumarto 2003), and in the crisis context, many asylum seekers experience increased and often acute isolation from legal systems, welfare systems, and community support structures due to state changes to a range of policies. Broadly, affected asylum seekers fall into three key groups: (1) those in transit, (2) those living in the community in host countries, and (3) those in immigration detention.

For asylum seekers in transit, movement typically becomes further restricted to the point where access to territory, and thus to asylum procedures, is almost impossible. Those asylum seekers already living in host communities can face exclusion from a range of welfare supports, and in many cases special measures that are available to citizens during crises are not available to asylum seekers, even in cases where they have been living and working in the community for many years. Crisis-driven restrictions on freedom of movement also prevent many from accessing important community supports on which they rely. During crises, asylum seekers in immigration detention often face increased health risks due to crowded conditions, lack of protective equipment, limited access to healthcare, and increased isolation due to restrictions on visits. (Singer et al. 2022).

Decisions made during times of crisis may not intend to cause harm, but their impact can be profound and lasting. The context in which decision makers make crisis-driven decisions, and the manner in which they articulate and justify these decisions, is important, as it reveals the priorities of societies and the extent to which certain actors are willing to advance their agendas. The terms “crisis,” “disaster,” and “emergency” are “closely interconnected, interdependent and overlap significantly,” and are often used interchangeably. Drawing on a review of a range of literature, AL-Dahash et al. (2016, p. 1193) show that crises typically feature uncertainty, unpredictability, present new and unexpected situations, and can threaten the basic assumptions of a system. Nevertheless, the authors identify no clear definition for a crisis beyond the review of definitions proposed by others, going only so far as to explain a crisis as an abnormal disruption which produces a high level of risk, and emphasize the close connection between crisis, disaster and emergency. While not the tight definition that might please some, their analysis does illustrate an important definitional challenge when analyzing crisis—when is a situation a crisis, and when is it something else? Writing in the context of clinical psychological intervention, Callahan (1994) argues against the interchangeability of “crisis” and “emergency,” identifying differences in the time aspects of each and the decision-making hierarchy. In this intervention context, an emergency is a serious, unexpected, and often dangerous situation requiring immediate action, whereas a crisis is a time of intense difficulty or danger which can extend over a longer period and require more systemic interventions.

This article draws examples from four recent crises of the past two decades that have a particular relevance for mobility and forced migration—(1) the response to the attacks of 11 September 2001 and the ongoing crisis in global security linked to the Global War on Terrorism; (2) the 2015–2016 migration “crisis” in Europe; (3) the COVID-19 pandemic from 2020; and (4) the Russian invasion of Ukraine in February 2022. These case studies are chosen for their visibility, their place in contemporary collective memory, and for their ongoing impact in terms of state response through law and policy, highlighted by the shaping of discourse around forced displacement, asylum, and the corresponding need for border protection. The attacks of September 11 are examined first as an example of how crisis and political opportunity can combine to produce otherwise unlikely policies that affect forced migrants. Second, the differing approaches to asylum seekers arriving in Europe in 2015–2016, then in 2022, are juxtaposed to draw out the situational nature of vulnerability and human rights, and the idea of the ideal vulnerable migrant. Third, the impact of restricted mobility during the COVID-19 pandemic on asylum seekers is used to show the manner in which restrictive measures designed to protect can harm already vulnerable groups of people, and introduce further elements of control to already heavily controlled populations. Drawing on these three case studies, the article concludes with a consideration of how states and other actors use vulnerability, and what role the vulnerability concept might play in present and future responses to crises.

To facilitate this analysis, the paper uses a combined method that engages the law, state policy, and the accompanying political rhetoric used to explain and justify that law and policy. Law is examined here to demonstrate the status quo, the normative structures that give impetus to state policies. In this paper, law provides an overarching context, but is not the primary point of analysis. Law is connected to policy, showing the particular manner in which states construct an environment that enhances asylum seeker vulnerability. By demonstrating the novel policy developments of states in times of crisis in various contexts over time, and how they connect and overlap, patterns of calculated exclusion are highlighted. Policy and law, in particular international law, are often not analogous, they can diverge with the varying interests of states. To show this divergence, and the manner in which it is explained by those who create and implement policy, this paper draws extensively on the political rhetoric used by various national and regional leaders to explain and justify their responses to the respective crises outlined above. Showing and examining the language used by these leaders, when analyzed alongside the prevailing law and policy, allows for valuable insight into how crisis is captured, framed and harnessed to drive policies that enhance vulnerability, and how crisis is used to justify, enable, and normalize exclusion of asylum seekers.

## Vulnerability Theory as an Approach to Crisis

The notion of vulnerability lacks any normative foundation or universally agreed definition; however, it arises and is applied regularly in legal and policy contexts (Beduschi 2017). Much has been theorized about the vulnerability concept since Martha Fineman’s initial seminal text on the topic. In this early exploration of a theory of human vulnerability, Fineman explained vulnerability as universal and constant, arguing for emphasis on both privilege and discrimination, and stressing the need for a responsive state in managing common vulnerabilities (Fineman 2008). Fineman has further refined and explained this approach by repeatedly stressing the need for a state that is responsive to vulnerability (Fineman 2010), and showing that while vulnerability is “universal and continuous,” overarching frameworks of equality are not sufficient to ensure “equitable treatment for differently positioned individuals” (Fineman 2017: 134–135). Others such as Turner (2006) and Morawa (2003) earlier focused on vulnerability specifically in terms of human rights, with the former identifying universal human vulnerability as a foundation for a framework of human rights, and the latter examining how and to what extent various international human rights tribunals assign vulnerability to particular groups.

Vulnerability can similarly serve as a divisive and exclusionary tool (Engström et al., 2022). Simply demonstrating some level of vulnerability may not be enough to gain the assistance needed to survive where a state is not sufficiently responsive, indeed in the asylum context a clear distinction between vulnerable and less vulnerable has emerged as a means of allocating state services (Sözer, 2020). Peroni and Timmer (2013:1085) show how despite the potential of the use of group vulnerability by courts to address substantive equality and move “towards a more inclusive universal human rights subject,” this form of reasoning brings the risk of essentialism, stigmatization, and paternalism. Indeed, as Rebecca Yeo (2020: 680) shows in her recent study of asylum seekers in the UK, the vulnerability label can obscure systemic oppression, leading to a “hegemonic acceptance that some people are worthy of support and others are not.”

This paper draws on a combination of the approaches outlined above, cognizant of the vexed nature of universal human vulnerability, and understanding vulnerability as situational, contextual, and individual, deeply linked to the capacity and willingness of the state to not simply respond to vulnerability, but to not generate and exacerbate it. By looking at state responses to crises through the lens of vulnerability, and the position of particular groups and individuals in relation to state actions in time of crisis, there is a clear pattern of enhanced vulnerability where states either fail to act or act in a manner detrimental to those already in vulnerable situations.

## Crisis as a Political Opportunity—USA and Australia Post-September 11

On September 11, 2001, four hijacked commercial airliners crashed into targets in the USA in a series of coordinated attacks. Two of the aircraft struck the World Trade Center towers in New York City, one hit the Pentagon in Virginia. The fourth, seemingly intended for a US government building in Washington D.C., was forced down by passengers in a field in Pennsylvania.

US President George Bush was in Florida when the attacks occurred, but he returned that evening to Washington D.C. to deliver a televised address from the Oval Office in the White House. Bush declared:

These acts of mass murder were intended to frighten our nation into chaos and retreat. But they have failed; our country is strong.

[…]

Today, our nation saw evil, the very worst of human nature. And we responded with the best of America -- with the daring of our rescue workers, with the caring for strangers and neighbors who came to give blood and help in any way they could.

[…]

I've directed the full resources of our intelligence and law enforcement communities to find those responsible and to bring them to justice. We will make no distinction between the terrorists who committed these acts and those who harbor them.

Immediately following the first attack, I implemented our government’s emergency response plans. Our military is powerful, and it's prepared. Our emergency teams are working in New York City and Washington, D.C. to help with local rescue efforts. (Bush 2001)

Bush uses clear and simple language, and offers his message of protection and resistance in the context of a newly emerged threat. He beckons a good people to respond to the worst of humanity, and to remain resolute and strong. Crisis, and the attempt to instill a state of crisis, is central to his message, as he casts the USA in the role of active responder, not passive victim.

Operation Enduring Freedom, the US-led operation to remove the Taliban regime in Afghanistan and destroy Osama Bin Laden’s terrorist network there, began on October 7, 2001. The Taliban’s effective control of Afghanistan ended within months; however, the war continued for two more decades, with the final US troops withdrawing on August 30, 2021. Captured fighters in the Global War on Terrorism, dressed in orange prison jumpsuits at Guantanamo Bay, Cuba, became synonymous with the US-led conflict over the following years. Bin Laden, the mastermind behind the attacks, remained at large until US forces located and killed him in Pakistan on May 2, 2011.

In late 2001 and 2002, owing to the ongoing sense of threat connected to the attacks of September 11, 2001, the US enacted wide-reaching legislative reform and restructuring of its government. The Uniting and Strengthening America by Providing Appropriate Tools Required to Intercept and Obstruct Terrorism (USA PATRIOT) Act of 2001, more commonly known as the Patriot Act, came into effect on October 26, 2001. The act’s long name is further instructive in terms of its statement of purpose: “An Act to deter and punish terrorist acts in the United States and across the globe, to enhance law enforcement investigatory tools, and for other purposes” (USA PATRIOT Act 2001). Among its key provisions, the Act expanded the ability of law enforcement to conduct surveillance, increased penalties for terrorist crimes, and expanded the list of activities qualifying for terrorism charges. The Act received widespread criticism at the time of its implementation due the wide scope of its powers, the lack of apparent oversight, the intrusion on civil liberties, and the implications for non-citizens (Whitehead and Aden 2002). Many of these increased surveillance powers would be conducted under the umbrella of the soon to be formed Department of Homeland Security. Under the pretext of response to crisis, significant changes were made to the ability of the state to intervene in the lives of its citizens, in particular through increased surveillance.

The now ubiquitous US Department of Homeland Security followed in 2003, in the largest reorganization of federal government since the creation of the Department of Defense in 1947. Created through the Homeland Security Act of 2002, the Department of Homeland Security is now the third largest US Cabinet agency with 250,000 employees and other personnel across more than 20 component agencies. The Department, according to its 2022 key priorities, “was established in the wake of the September 11th attacks with the core responsibility to keep our nation safe” (DHS 2022). As with the Patriot Act, crisis-driven policy was allowed to prioritize perceptions of lessened state security over the rights of citizens.

While the above measures certainly affected how non-citizens were treated and viewed, both in a legal and social sense, new measures also had a direct impact on mobility. Discussion of vulnerability was, however, largely absent in debates that presented terrorists and victims as mutually exclusive elements of the broader global response though the war on terror. Where discussion of vulnerability was present, it was in the context of vulnerability to terrorism, not any perceived vulnerability of terrorist actors themselves (Mitchell 2003). The Aviation and Transportation Security Act (ATSA), enacted on November 19, 2001, established the Transportation Security Administration (TSA), which presides over all the security of transport inside and to the USA. TSA operates as an agency of the Department of Homeland Security, and defines its mission as “Protect the nation’s transportation systems to ensure freedom of movement for people and commerce” (TSA 2022). ATSA also provided for new and more intensified security measures that are now commonplace: identity checks and extensive security screening at airport terminals. David Schaper, writing on the legacy of the attacks 20 years on, shows the difference between a time of minimal airport security and now: “Now, travelers often stand in long lines at security checkpoints with wait times that can exceed an hour. We take off our shoes, empty our pockets and take laptops and other devices out of carry-on bags before stepping into high-resolution, full-body scanners, while our bags go through 3D-imaging X-ray machines. And don't forget to take your liquids of 3.4 oz or less out of your carry-on” (Schaper 2001). With increased and fully centralized security comes the capacity for greater surveillance and restrictions on movement, as well as the perception of safety, an assurance that the state is acting in its role as protector. It is an opportunity to monitor who moves within a territory, and importantly, who can access that territory in the first place.

Arguably none of this would have been possible, at least not in such a manner, were President Bush and his administration not emboldened by the crisis that surrounded them. Crucially, following the attacks of September 11 Bush enjoyed a huge surge in popularity. In answer to the question “Do you approve or disapprove of the way George W. Bush is handling his job as president?”, in the period from September 7 to September 10, 2001, only 51% of those polled by Gallup responded that they approved. The next poll, from September 14 to September 15, saw that figure increase to 86, and to 90 on September 21 to September 22. Bush’s approval rating did not drop below 75 until June 2002 (Gallup 2022). The majority of the measures outlined above, enacted in times of crisis and with an assumption (even a promise) of temporariness, remain present today. The shift in global security discourse following the attacks, including the function of border control in managing migration, remains central to the deterrence policies of wealthy states as they maintain the use of measures grounded in crisis decision-making to justify their present policies.

Another leader present in Washington on September 11, 2001, by coincidence, was incumbent Australian Prime Minister John Howard. Howard was behind in the polls and facing defeat in the election scheduled for later that year. Less than 1 month earlier, in a defining moment for Howard, the Australian government had refused entry to 438 rescued asylum seekers aboard Norwegian freighter *MV Tampa*, following an extended standoff in the waters to Australia’s northwest (Marr and Wilkinson 2003). The incident opened the door for Australia’s new migration policy, known as the Pacific Solution, under which unauthorized maritime asylum seeker arrivals would be taken to processing facilities on Nauru and Manus Island (Papua New Guinea) rather than to Australia. Howard was resolute in his motivation for refusing entry to those aboard the Tampa: “I believe it is in Australia’s national interest that we draw a line on what is increasingly becoming an uncontrollable number of illegal arrivals in this country” (NMA 2022). Following the attacks of September 11 national security, fused with border security linked to asylum seeker arrivals, became a key election issue. At the launch of his party’s election campaign on October 28, 2001, Howard declared:

So therefore a military response and wise diplomacy and a steady hand on the helm are needed to guide Australia through those very difficult circumstances. National security is therefore about a proper response to terrorism. It’s also about having a far sighted, strong, well thought out defence policy. It is also about having an uncompromising view about the fundamental right of this country to protect its borders. It’s about this nation saying to the world we are a generous open hearted people taking more refugees on a per capita basis than any nation except Canada, we have a proud record of welcoming people from 140 different nations.

But we will decide who comes to this country and the circumstances in which they come.

[…]

We have had a single irrevocable view on this, and that is that we will defend our borders and we’ll decide who comes to this country. But we’ll do that within the framework of the decency for which Australians have always been renowned. (Howard 2001)

Like Bush, speaking on September 11, Howard’s language is direct, evoking images of threat, resistance, and protection. Following the election on November 10, 2001, Howard’s Liberal-National coalition retained office with an increased majority, and the opposition Australian Labor Party recorded its lowest primary vote since 1934. Howard’s border policies, born from and solidified during a period of crisis, remain largely unchanged to the present day and enjoy bipartisan majority support in Australia’s parliament. Like the reforms in the USA following the attacks of September 11, many of which directly affect who can move, when, where and how, Australia’s offshore asylum seeker solution is engrained and largely unchallenged. This parallel is significant, as it was under the shadow of the attacks of September 11 that Howard successfully implemented his new policy. Any challenges to the new migration regime under Howard were countered by the imperative of protecting Australia’s borders in the context of the uncertain environment of the Global War on Terror. These policies were justified as necessary to protect Australia from new and unknown threats in challenging times (Holland 2010).

## Some are More Vulnerable than Others—the European Migrant “Crisis” of 2015–2016 and the Mass Displacement from Ukraine in 2022

In 2015, European Union (EU) member states received 1,216,860 first-time asylum applications, an increase from 530,560 in 2014 and 338,190 in 2013. 1,166,815 applications were received in 2016, dropping to 620,265 in 2017. The total number of first-time asylum applications has remained consistent since 2017 (EUROSTAT 2022). The majority of these arrivals were from Syria, Afghanistan, and Iraq, and were male. Greece received more than 850,000 arrivals by sea in 2015, the majority of whom moved towards Northern and Western Europe through the Western Balkans. Turkey remained the largest refugee hosting country in the world, registering over 2.5 million Syrians in 2015 alone. Notably, the United Nations High Commissioner for Refugees also listed eastern Ukraine as an area of concern following the beginning of the Russo–Ukrainian war in 2014. UNHCR regarded the situation as “precarious, with largescale displacement challenging resources and diminishing the resilience of both internally displaced people (IDPs) and host communities” (Spindler 2015).

The state response to this rapid surge in asylum seeker arrivals to Europe in 2015–2016, for the most part, was a series of restrictive measures designed to prevent access to territory and to make the asylum seeking experience so difficult as to discourage those already present and deter those who might think to come. States employ a range of measures to deter potential arrivals. Non-admission policies, such as the EU–Turkey deal, whereby asylum seekers arriving in Greece are not processed there but are instead transferred to Turkey, limit access to asylum procedures. Non-arrival measures including carrier sanctions, visa regimes, and interdiction prevent access to the territory of asylum states. Offshore asylum processing and relocation of refugees to third countries further ensure physical and legal barriers to asylum, and have recently been pursued by Denmark and the UK following their observance of Australia’s equivalent policy over the past two decades. Criminalization of irregular migration and human smuggling plays a further deterrent role, as do indirect deterrence measures intended to make the asylum country less attractive (Gammeltoft-Hansen and Tan 2017). These are now standard tools used by European states to repel unwanted asylum seekers (Ghezelbash 2018; FitzGerald 2019; Cantor et al. 2022).

The following words by then European Commission President Jean-Claude Juncker, on October 13, 2016, are instructive in terms of the light they shed on the European response to this “crisis,” and the European self-image that the required response to the crisis sought to uphold.

When crisis came, it put extreme pressure on our system, and it found our weakness. Like water against a dam, it found the gaps and the cracks. It put our very foundation to the test.

[…]

As each crisis unfolded – by the day, by the hour – the European Union acted as a crisis manager. We proposed solutions, we mobilised resources, and we helped to build bridges where solidarity was missing.

[…]

During the first year of the refugee crisis, we proposed and implemented a comprehensive migration agenda: saving lives at sea, providing humanitarian aid, supporting our Member States most under strain, relocating asylum seekers across the Union and returning irregular migrants. A lot has to be done in that respect, by the way.

But this brings me to the critical moment. As refugees continued to risk their lives on the sea, we had to make a breakthrough. We saw that incremental change was not enough. We decided that our next move had to be bold.

In the first six months of this year, we proposed the European Border and Coast Guard; we signed the EU-Turkey statement; we launched a fundamental reform of our asylum system; and we proposed new Migration Partnerships with countries in Africa (Junker 2016).

Junker presents a Europe emboldened by crisis, solidified in its union. Europe acts decisively and boldly to prevent both threats to the union and preserve the lives of those migrants who might otherwise perish in their attempts to enter it. Nevertheless, the measures he lists do not so easily fit alongside their purported humanitarian motivations. The European Border and Coast Guard has been accused of involvement of illegal pushbacks in the Mediterranean and on the Poland-Belarus border; the EU–Turkey statement was made in clouded circumstances and is rife with problematic elements; reform of the asylum system does not prioritize protection; and partnerships with African countries raise a range of human rights issues, including focus on refugee containment, not protection.

The EU–Turkey statement in particular is a curious point of focus for Junker. The statement provided that:

1. All new irregular migrants crossing from Turkey into Greek islands as from March 20, 2016 will be returned to Turkey.
2. For every Syrian being returned to Turkey from Greek islands, another Syrian will be resettled from Turkey to the EU taking into account the UN Vulnerability Criteria.
3. Turkey will take any necessary measures to prevent new sea or land routes for illegal migration opening from Turkey to the EU, and will cooperate with neighboring states as well as the EU to this effect.

The provision for “any necessary measures” is particularly unclear, and seemingly relinquishes any control from the EU to Turkey. Turkey, for its part, would receive improved visa access to the EU for its citizens, and 6 billion EUR in support for Turkey to provide assistance for refugees present in Turkey (European Commission 2016). The EU’s choice of partner in deflecting asylum seekers from its borders has received widespread criticism. Amnesty International described 2016 as Europe’s “Year of Shame,” and Imogen Sudbury from the International Rescue Committee (IRC), speaking 5 years on from the conclusion of the agreement, called the agreement “a stain on the European Union’s human rights record,” one for which those in need of protection “continue to pay the price” (Amnesty International 2017; Sudbury 2021). Indeed, despite its initial implementation as “a temporary and extraordinary measure which is necessary to end the human suffering and restore public order,” the deal remains in place with an increase in funding (European Commission 2016). Particularly problematic in terms of the rights of vulnerable migrants are the assumption that Turkey is a safe third county for asylum seekers, the lack of procedural safeguards in Turkey due to it not being subject to EU law, and Turkey’s poor record of according asylum claimants and refugees proper access to asylum procedures (Poon 2016). Evidence from Turkey that many asylum seekers returned under the EU–Turkey deal are detained, and many risk onward deportation without access to legal aid and international protection, suggests that these fears are well founded (Tunaboylu and Alpes 2017). Institutional progress in recognizing vulnerability has been slow, and according to the European Council on Refugees and Exiles (ECRE) “it is the Court’s [European Court of Human Rights] recognition of the particular vulnerability of certain applicants for international protection rather than the intrinsic vulnerability of asylum seekers as a vulnerable group per se, which has been translated into EU and national legislation.” (ECRE 2017).

The experience of asylum seekers arriving from the Middle East in 2015–2016 and those entering the EU from Ukraine in 2022 exist in sharp contrast. On February 24, 2022, following a period of intense military build-up of Russian forces around Ukraine’s borders, Russia invaded Ukraine in an intensification of the Russo–Ukrainian war that began in 2014. In the weeks since the invasion approximately 5.2 million Ukrainians have fled the conflict to other countries, leading to the largest mass movement of refugees in Europe since the Second World War. Millions more are internally displaced within Ukraine. The vast majority of those fleeing Ukraine, estimated at 90%, are women and children, with men of conscription age (18–60 years of age) not allowed to leave Ukraine (UNHCR 2022). Numerous reports also document difficulties faced by foreign nationals attempting to leave, both in accessing transport to the border and at the border itself. Reports include priority given to white migrants on transport and at border crossings, measures to force people of African descent to the back of queues, racialized denial of entry to some neighboring countries, and restricted visa access for people of African descent. These reports saw the United Nations Working Group of Experts on People of African Descent, the Special Rapporteur on contemporary forms of racism, racial discrimination, xenophobia and related intolerance, and the Special Rapporteur on the human rights of migrants reaffirm in a statement that:

It is essential that equal treatment is upheld for all, including people of African descent seeking to depart from Ukraine. This includes by agents of the State, especially border officials. We wish to restate the recent call by our multiple mandates to respect and protect the fundamental rights and freedoms of all persons affected by the armed conflict. (OHCHR 2022)

Even in a case of an urgent border crossing to flee a rapidly escalating conflict, not all people attempting to move experience the same opportunities; some are more vulnerable, more wanted, than others, seen as somehow more deserving of support and the chance to flee.

On the institutional level, the EU was remarkably swift to act in response to the millions of Ukrainians entering through its borders. Noticeably, as da Lomba and Vermeylen (in this issue) observe, very little vulnerability argumentation has emerged in the response to this new “crisis.” The resultant question of when is vulnerability argumentation needed is discussed in depth by Engström et al. (2022), who argue that vulnerability reasoning is used when there is a desire to engage in “selectivity and prioritisation,” which as they show can easily become “exclusion and politicisation.” By March 4, 2022, the EU had enacted the Temporary Protection Directive, giving immediate protection and legal status to those fleeing Ukraine. No similar steps were taken for Syrians or others in 2015–2016. Speaking following the announcement, European Commission Vice-President for Promoting our European Way of Life, Margaritis Schinas said:

The unprecedented decision to grant immediate protection to all those who call Ukraine their home is now being translated into practice. To help make this process as smooth as possible, the Commission is supporting Member States with operational guidance. For example, to ensure people can move around the Union unhindered, we clarify that they should be able to receive 15 day visas at the border and that in any case carriers should not be fined for transporting them without documentation. (European Commission 2022)

Commissioner for Home Affairs, Ylva Johansson, expressed similar sentiments of support:

In a matter of days, 3 million people crossed into the EU. The show of solidarity has been immense and the reaction of the authorities impressive; but real challenges exist to ensure national systems do not become overwhelmed and that people enjoy the protection they deserve. (European Commission 2022)

Two of the fundamental elements for restricting movement of people, visas and carrier sanctions, were swiftly set aside in the case of Ukrainian refugees. Forced migrants of other nationalities who attempt to enter Europe undocumented are considered “illegals,” and those who transport them are “smugglers” and “traffickers,” profiting from illegal activity. The migrants are in many cases repelled en route by border guards. A migrant from Ukraine arriving with no passport or visa, transported by unregulated carriers, gains entry and temporary protected status, “the protection they deserve.” Solidarity, evident both here and in 2015–2016, has taken on a different meaning. Solidarity in 2015–2016 referred to the need for a coordinated response to an unwanted surge in asylum seeker arrivals, not to the asylum seekers themselves, who were blocked from entry and diverted to Turkey and elsewhere. In this situation, the threat was external and solidarity projected inwards, inside the EU’s borders. Solidarity in 2022 means solidarity towards Ukraine, refugees in need of protection from a conflict on the European continent. Solidarity here projects outwards, beyond the EU border, in a display of welcome but also of resistance and strength. Solidarity, like vulnerability, means different things depending on the context—vulnerability of Europe to a threat, vulnerability of Ukrainian refugees; solidarity within Europe, solidarity with the victims of a common enemy.

Who deserves what, who is more deserving, and whom states and communities feel obligated to assist connects to a range of factors. Vulnerability, or perceived vulnerability, plays a central role. Vulnerability is a commonly invoked term that lacks any clear definition in international law, and that is used to categorize and protect but can also be used to exclude. Being labeled as vulnerable can provide a path to access to rights and services, but just as those who are declared vulnerable benefit from this label, those who are not seen as vulnerable—or in many cases, not vulnerable enough—are sidelined from the protections that this label can offer. As Sözer (2020, p. 2163) shows, it has become morally acceptable and desirable to assist only certain categories of forced migrants, so that who is “vulnerable” is “an unevenly distributed label.”

There are certain categories of persons who typically feature in notions of what may constitute vulnerability in the migration context, for example, unaccompanied minors, children, families, women, those with serious health conditions, or those with disabilities. Very rarely do single men fit easily into this category, yet in many contexts they make up a large percentage of asylum seekers, and can most certainly be vulnerable given their circumstances. This gendered element of asylum seeker vulnerability is especially evident in the examples given above, where in 2015–2016 the majority of those seeking protection in Europe were men, and those fleeing Ukraine mostly women.

Men in this case often fit into a separate category of vulnerable persons, those whose lack of apparent vulnerability increases and enhances their vulnerability, and leads to problems that may not have manifested were they seen as vulnerable enough in the first instance. A person who is not vulnerable enough today, left without access to services, will in all likelihood be vulnerable enough in a week, a month, or a year. Male refugees are typically more likely to be perceived as a threat, both due to their racialization and due to the connection of young Middle Eastern and African men with the war on terror. They are also likely to elicit less sympathy due to their age, a response often underpinned by the assumption that as men of military age they should remain in their countries to fight, not leave to seek refuge (Szczepanik 2016:25–26). This problem is avoided in the Ukrainian case by the almost total lack of male Ukrainian refugees of military age. The result is a group of asylum seekers that is often perceived as being in less need than others, and that is not prioritized for access to support services where they exist. This is driven both by a scarcity of resources in many cases, as well as traditionally held ideas of male strength and resilience.

This denial of access to services typically results in poverty, and is the source of enhanced vulnerability when comparing different groups of asylum seekers across different and overlapping crises. Poverty is understood and defined in various ways, and like vulnerability there is no settled international legal definition of exactly what the term means. Broadly speaking, poverty refers to a state where a person’s income is so low that their basic needs for daily living cannot be met. More fundamentally, poverty is grounded in “being able to live a life in dignity and enjoy basic human rights and freedoms” (OHCHR, 2006). Through the denial of access to services, and the condition of poverty that can often result, states are able to place asylum seekers in a situation that leaves them with very few options to meet their basic needs, and they must rely on informal support networks that can vary significantly in their capacity to provide support (Phillips 2018). While not as obvious as intercepting boats or immigration detention, this enforced state of poverty is also part of the overall deterrence approach of states for preventing unwanted migration—it sends a clear message that you may try to come, but if you do succeed in coming we will make even your day to day survival a struggle.

The action by the state is the denial of access to services, and the result is a state of poverty for those who have no other means to support themselves. So while in a rights sense the focus here is on poverty, poverty is both the consequence and the intent of the action. Poverty emerges as the consequence of systematic deprivation of the means to survive in dignity, both in terms of lack of access to support services from the state, but also from the capacity to support oneself by preventing access to the labor market through employment restrictions. Thus, even where work may be available it is prohibited, and the only other forms of state support, in the form of welfare assistance and other programs, are reserved for those deemed more vulnerable.

In the case of migration, it is important to note that in most countries where migrants and asylum seekers find themselves in conditions of poverty, there are also many citizens and residents who are similarly unable to provide for their own basic needs. So, what makes the situation of an asylum seeker different from citizen who experiences poverty? Is there a particular added layer of vulnerability experienced by asylum seekers? Two key elements here are permanence and security. An asylum seeker with no permanent or secure status remains at the whim of the state in which they have sought refuge, bringing with it an enhanced vulnerability driven by a lack of security of place that citizens are less likely to experience. This lack of security of place may manifest in areas such as vulnerability to labor exploitation, no access to secure housing, barriers to health care, and a lack of legal certainty. For example, as Sormunen (in this issue) shows, the lack of access to legal remedies can be a source of enhanced vulnerability.

Poverty in this context is viewed as a consequence of a denial of access to services that are more readily available to either citizens, residents, or other categories of asylum seekers. Poverty is the condition that results from this denial; hence, the choice to examine the outcome through the poverty lens in terms of international human rights law. This is where tools are available to address the human rights element of denial of access to services.

While there is no clear definition of poverty in international law, some guidance comes from the United Nations Committee on Economic, Social and Cultural Rights (CESCR), which sets forth that.

In the light of the International Bill of Rights, poverty may be defined as a human condition characterized by sustained or chronic deprivation of the resources, capabilities, choices, security and power necessary for the enjoyment of an adequate standard of living and other civil, cultural, economic, political and social rights. (CESCR 2001)

Drawing directly on the International Covenant on Economic, Social and Cultural Rights (ICESCR), the rights to work, an adequate standard of living, housing, food, health, and education all have a direct bearing on poverty eradication (ICESCR 1966). Beyond the ICESCR, the CESCR also maintains that “all civil and political rights, as well as the right to development, are also indispensable to those living in poverty” (CESCR 2001). Justiciability, however, remains an ongoing challenge—some human rights are more justiciable than others, and many of the rights that are more directly connected with poverty are significantly less justiciable. (Nanda 2022).

To what extent then is there compatibility of this state approach of denial of services with international human rights law? Is the denial of support services that leads to poverty in conflict with international human rights law? If a right to live free from poverty is established then perhaps yes. Hence, the utility of framing this as a legal issue in terms of poverty, not as access to services in a direct sense. The European Court of Human Rights ruled in the case of *M.S.S. v. Belgium and Greece* (2011) that conditions for asylum seekers in Greece were so bad that not only had Greece breached the European Convention on Human Rights on account of the poor conditions, but Belgium had too by virtue of transferring an asylum seeker to Greece. This, however, is an extreme case, and not so helpful when looking at the cumulative effect of state actions and inactions.

What is problematic in this case for human rights law is the pattern, intent, and outcome, not necessarily the act. This is equally the case for the use of deterrence that leads to *refoulement*—it may not be sole individual measures that challenge human rights law, but there is a pattern, intent, and outcome that may lead to a situation where there is a more easily identifiable breach (e.g., *refoulement*). Proving the intent of the state remains difficult, however. For example, unless a state actually sends a person across a frontier when does an action become *refoulement*, what level of action is strong enough to constitute *refoulement*? Is it enough to create a hostile and coercive environment where asylum seekers no longer wish to live? If a person ultimately leaves due to poor living conditions, purportedly of their own volition, or another person decides, based on the conditions in a particular country, not to go there to seek asylum, does this fall under the *non-refoulement* provision of the 1951 Convention Relating to the Status of Refugees (“Refugee Convention”)? Without establishing pattern, intent, and outcome, it can be very difficult to determine the extent to which state actions are designed to cause harm. Outcome and pattern are perhaps easier to identify, they are often more explicit and draw more attention; intent is not so easy, yet it is essential to prove the motivation for a course of action. If enhanced vulnerability is an unintended outcome of a policy, the policy may have been poorly formulated and executed; if this vulnerability is an intended consequence, however, designed to test and break resilience, the policy’s very legitimacy is at stake.

The intersection of poverty and vulnerability in the case of asylum seekers comprises many elements, and may change dramatically depending on the individual or group. What is clear is that denial of access to services makes enhanced vulnerability through poverty more likely, and what services or protection an individual can access vary based on the perception of an individual by those controlling access to the required services. If all asylum seekers are vulnerable, some are certainly more vulnerable than others, and a lack of perceived vulnerability, paradoxically, can lead to enhanced vulnerability when support is not forthcoming.

## COVID-19—Restrictions that Reduce Mobility and Enhance Vulnerability

While the outbreak of war in Europe has taken much of the attention of the public and decision makers away from the COVID-19 pandemic, the impact of measures enacted to prevent the spread of the virus remain, and are felt in particularly by those in already vulnerable situations.

COVID-19 has brought about wide-reaching changes in how migration is managed. Many borders were fully closed for extended periods, others made difficult to cross, and most are subject to greater levels of surveillance than ever before. Even as the pandemic recedes in many parts of the world increased border control measures such as vaccines and health tests are required for travel. In this context, asylum for a period effectively ceased to exist, and it is as yet unclear to what extent these COVID-inspired changes will continue to impact access to territory by asylum seekers as the pandemic continues to cast its shadow over global mobility. This uncertain future, which is potentially paradigm changing, has a significant impact on forced migration research as it adds additional layers to how states prevent access to territory by asylum seekers. One developing hypothesis is that many of the COVID-19-driven changes in migration management and control that have been introduced during the pandemic will not be rolled back once the pandemic has receded. This has deep and ongoing implications for human rights law, refugee law, and the right to asylum, and requires further study to fully comprehend its impact.

There is an emerging discussion among refugee law scholars, such as Ghezelbash and Tan (2020), concerning the future of asylum post-COVID-19, where they show how the COVID-19 pandemic has all but extinguished the right to seek asylum in the Global North. Their arguments offer a starting point for considering how deterrence of asylum seekers may function as the COVID-19 crisis is better understood, and what this means for how deterrence and human rights are likely to shape one another. If migration, and in particular, asylum, are to take a dramatically different direction after the COVID-19 pandemic, it is essential that scholarship on migration and asylum is fully aware of these changes, and is responsive to them.

A common response by states to those seeking to access to their territories for the purpose of seeking asylum, access which is typically unwanted, is to find ways to prevent this access. It is well established that states mobilize significant resources to prevent access to their territories and to asylum, and that there are various measures that states employ to achieve this goal. Such practices have variously been described as “repulsion” or “deterrence,” and located within what has been called a “deterrence paradigm” (Gammeltoft-Hansen and Hathaway 2015; Gammeltoft-Hansen and Tan 2017; FitzGerald 2019). A range of authors have shown that a complex system of deterrence measures which states employ to prevent any contact by refugees with the territory of the receiving state exists, and that states’ commitments to international law are often far more present in their rhetoric than in their practice (Gammeltoft-Hansen and Hathaway 2015; Gammeltoft-Hansen and Tan 2017; Ghezelbash 2018; FitzGerald 2019, Cantor et al. 2022). For more than 2 years, many of these measures were not fully in action as borders were completely closed, or at least much more tightly controlled, due to changes implemented in the context of state responses to COVID-19. As restrictions lift and mobility gradually begins to increase, it is uncertain what the impact will be on those seeking asylum, in particular what level of access to territory they will enjoy. It is possible that previous restrictive measures are intensified, COVID-19 restrictions are not lifted (at least fully), or that new restrictive measures are created.

If we are indeed looking at the end of territorial asylum, and borders are to be further restricted and monitored, we need to think about how to understand these changes in both a practical and a conceptual sense. States will change and adapt their behaviors as the prevailing political context allows, and international law provides structures, but not boundaries, to state actions. Furthermore, state practice shows that state interest typically trumps strict observance of international legal norms. This new COVID-19 context has opened up a range of possibilities for states to further frustrate access to asylum, and even as many restrictions are removed they will be removed for some but not for others. States showed a willingness to target measures towards particular countries, for example, the EU entry ban on southern African countries, and there is nothing to suggest that similar targeted measures will not be implemented in the future in controlling unwanted migration.

The impact of COVID-19 has been felt across industries and has led to job losses and layoffs in many sectors. Social systems have come under immense pressure, and governments have been forced to respond to a large number of people without any form of income or savings. Many countries have provided special benefits to workers and entrepreneurs to assist them to manage during the pandemic, and to ensure that they will be able to recover once the pandemic has begun to recede. (ILO 2022) This support, however, has not always been available for all residents—Australia, for example, restricted access to income support to citizens and permanent residents, not temporary visa holders including asylum seekers (Whiteford 2020). This exclusion from support and services has led to an increase in vulnerability in already vulnerable populations. During lockdowns asylum seekers, often living in high-density, low-quality housing, have experienced fear of risk of infection, mental health concerns, poor nutrition, and problems accessing childcare, healthcare, and other services. In some cases, asylum seekers have been excluded from government financial support packages for those who have lost their livelihoods (Whiteford 2020). This compounding vulnerability leads to increased risk of further deterioration of living situations in the short term, and in the long term is likely to lead to further issues that may require state intervention and resources.

A particularly vulnerable group during the COVID-19 pandemic has been asylum seekers in immigration detention. Tensions at the infamous Moria camp on Lesbos in Greece linked to camp conditions, exacerbated by fears of a virus outbreak in the overcrowded camp at more than four times capacity, led to fires, protests, and the eventual closure of the camp (Gordon and Larsen 2021). Detention is widespread in the immigration context. It has become commonly used as a measure of first resort by many states as they seek to control unwanted migration. Many argue that it is used not only as an administrative measure, but also as a punitive one (García Hernández 2014; Vogl et al. 2021). There is a lot of work on conditions and use of detention, but less on who is and is not detainable, and why (De Genova 2016). Many of the existing studies focus on detention of groups that are seen as particularly vulnerable, such as children or unaccompanied minors (e.g., Mares et al. 2002; Farmer 2013; Smyth 2013; Australian Human Rights Commission 2014; Human Rights Watch 2016; Del Gaudio and Phillips 2018). The vulnerability of some groups is not contested, for example, children, pregnant women, the elderly, people with disabilities, and the very poor. This leads to a tension with the idea of “universal human vulnerability.” What about those who are not typically recognized as vulnerable? In the immigration context those with lower levels of perceived vulnerability are often more detainable, making them more easily isolated, excluded, and made invisible. Just as some are more vulnerable than others, so too are some more detainable. In the UK and Australia, for example, as many as 90% of immigration detainees are male (UK Government 2022; Australian Government 2023).

New pandemic realities brought increased risks for detainees—similar staffing and close contact conditions exist in immigration detention facilities as exist in aged care. Facilities may lack alcohol-based hand sanitizer (sometimes forbidden as contraband), and it may be difficult to socially distance (Singer et al. 2022). This increase in risk has not seen a shift to less restrictive options in the community for detainees who do not pose security risks. Rather, in Australia, the remote Christmas Island detention center was reopened, bringing further risk due to distance from specialized health care (Australian Human Rights Commission 2021). In fitting with the deterrence message, releases of detainees into the community were explained in economic terms, not because of the health and safety of the detainees. This leads to compounding vulnerabilities: asylum seeker status + detainability + increased risk due to pandemic.

As discussed above, it can often be difficult for those with less perceived vulnerability to demonstrate their vulnerability and to be seen as vulnerable—in particular, single men of military age are far more likely to be constructed and viewed as a threat than as vulnerable (Szczepanik, 2016). This notion of perceived threat rather than perceived vulnerability is central to their increased detainability. Increased detainability leads to a connected lack of access to services. When compared to those living in the community, detainees may lack access to family and social support, community support, health care, mental health services, and legal services, which has the effect of increasing their lived vulnerability (as opposed to their perceived vulnerability).

Paradoxically, as observed also above, it then may be the very lack of perceived vulnerability that makes a person highly vulnerable in an immigration context where detention is used as a routine tool of control, and even punishment. Through their exclusion and denial of access to services they are placed in a position of increased vulnerability, to the point that those with a higher level of perceived vulnerability have a far less damaging experience with the detention or expulsion apparatus (Phillips 2018).

This use of a particular deterrence measure on some groups more than on others fits neatly into ideas of securitization of migration, and challenges the utility of a vulnerability model that can be used to divide and exclude. This division and exclusion is achieved in the following ways: (1) It blocks access to asylum by strengthening systems of repulsion of unwanted migrants. The use of certain measures, especially detention, sends a strong security message. (2) It creates a problem and a threat, and offers a solution and a defense. (3) It creates physical divisions, those inside and those outside of the community or detention environment, those who are vulnerable and must be protected, and those who are detainable and can easily be excluded. (4) In times of crisis and uncertainty policies affecting vulnerable, excluded groups are likely to escape scrutiny, leading to increased risk of further vulnerabilization.

## Conclusion

Crises can be maximized by states and other actors to pursue policy objectives that might not be possible under normal circumstances. Political advantage can be gained, and policies that exclude groups such as asylum seekers in the name of countering an external or even existential threat, initially brought in as temporary measures, can become embedded. In times of crisis groups of migrants, variously or even similarly vulnerable, can be constructed either as threats or as worthy of support. Some, such as those now fleeing Ukraine, are embraced, while those fleeing Syria in 2015–2016 were largely repelled. A global pandemic provided the opportunity to secure borders and control movement more than at any point in recent history, and there is yet no certainty of the extent to which restrictive measures imposed to protect public health will be dismantled. Those living in already vulnerable situations experienced an enhanced level of vulnerability because of pandemic-related measures, with many experiencing further exclusion from an already excluded position. Crises provide both opportunity and threat, and the consequences for some are particularly damaging—future crises will likely present similar challenges for vulnerability, human rights, and access to mobility.
